# Supplementary material for: Collaborative nowcasting of COVID-19 hospitalization incidences in Germany
Source: PLoS Comput Biol. 2023 Aug 11;19(8):e1011394. doi: 10.1371/journal.pcbi.1011394 (PMC10446237; doi:10.1371/journal.pcbi.1011394)
Supplement: S1 Appendix — Additional figures, explanations, and in-depth information. (PDF) [file pcbi.1011394.s001.pdf]

Collaborative nowcasting of COVID-19 hospitalization incidences in Germany – S1 Appendix

Daniel Wolffram[1, 2], Sam Abbott[3, 4], Matthias an der Heiden[5], Sebastian Funk[3, 4], Felix Günther[6], Davide Hailer[1], Stefan Heyder[7], Thomas Hotz[7], Jan van de Kassteele[8], Helmut Küchenhoff[9, 10], Sören Müller-Hansen[11], Diellë Syliqi[9], Alexander Ullrich[5], Maximilian Weigert[9, 10], Melanie Schienle[1, 2], and Johannes Bracher[1, 2]

[1] *Chair of Statistical Methods and Econometrics, Karlsruhe Institute of Technology (KIT), Karlsruhe, Germany*
[2] *Computational Statistics Group, Heidelberg Institute for Theoretical Studies (HITS), Heidelberg, Germany*
[3] *Department of Infectious Disease Epidemiology, London School of Hygiene & Tropical Medicine, London, UK*
[4] *Centre for Mathematical Modelling of Infectious Diseases, London School of Hygiene & Tropical Medicine, London, UK*
[5] *Robert Koch Institute, Berlin, Germany*
[6] *Department of Mathematics, Stockholm University, Stockholm, Sweden*
[7] *Institute of Mathematics, Technische Universität Ilmenau, Ilmenau, Germany*
[8] *Center for Infectious Disease Control, National Institute for Public Health and the Environment (RIVM), Bilthoven, the Netherlands*
[9] *Statistical Consulting Unit StaBLab, Department of Statistics, Ludwig Maximilian University of Munich, Munich, Germany*
[10] *Munich Center for Machine Learning (MCML), Munich, Germany*
[11] *Süddeutsche Zeitung, Munich, Germany*

A Supplementary Figures

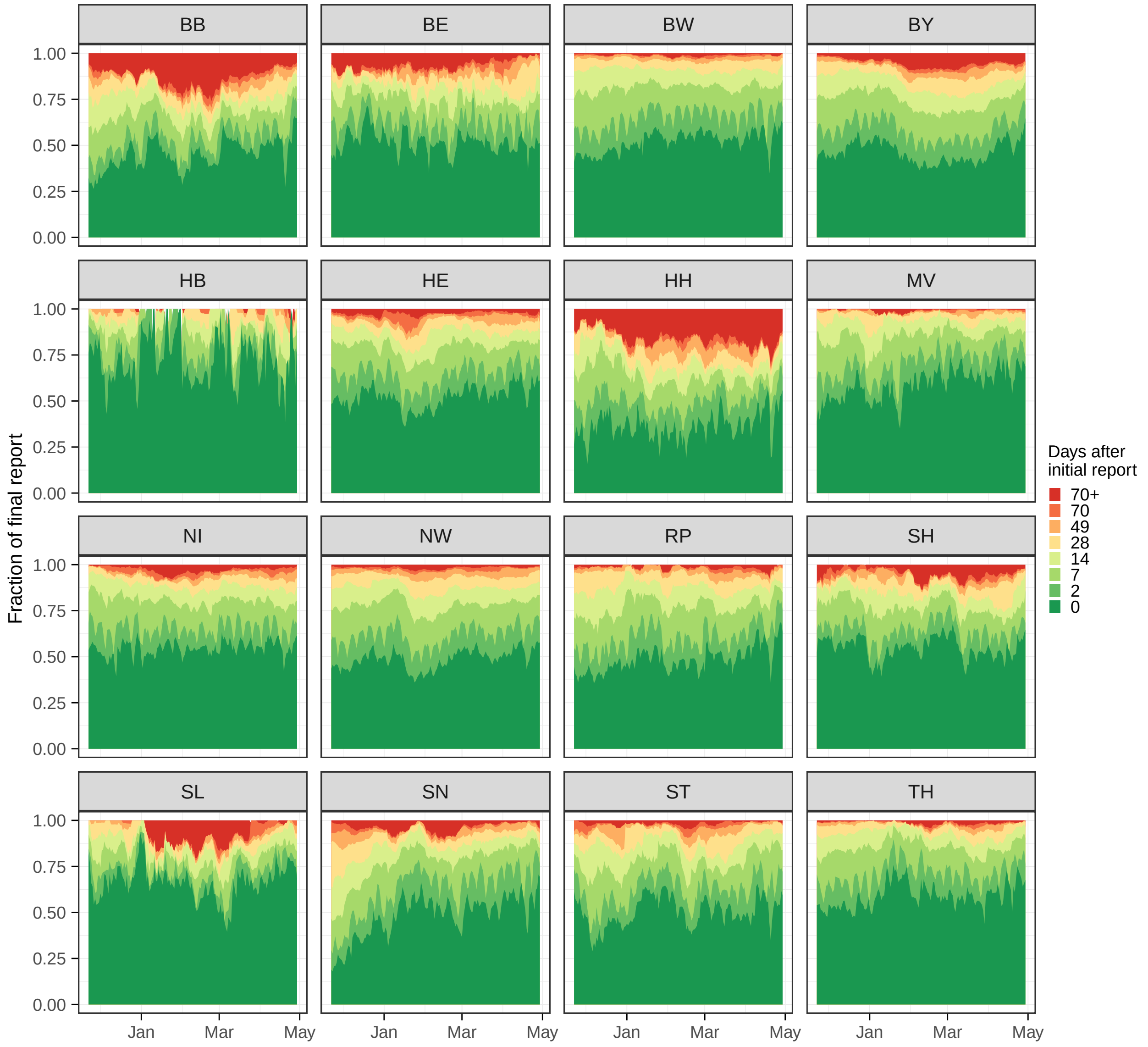

Fig A1: **Temporal development of the reporting completeness in the 16 German states.** Shown is the reported fraction of the final 7-day hospitalization incidences 0 to 70 days after the respective reference date. Abbreviations of federal states: BB = Brandenburg, BE = Berlin, BW = Baden-Württemberg, BY = Bavaria, HB = Bremen, HE = Hessen, HH = Hamburg, MV = Mecklenburg-Vorpommern, NI = Lower Saxony, NW = North Rhine-Westphalie, RP = Rhineland Pallatinate, SH = Schleswig Holstein, SL = Saarland, SN = Saxony, ST = Saxony Anhalt, TH = Thuringia.

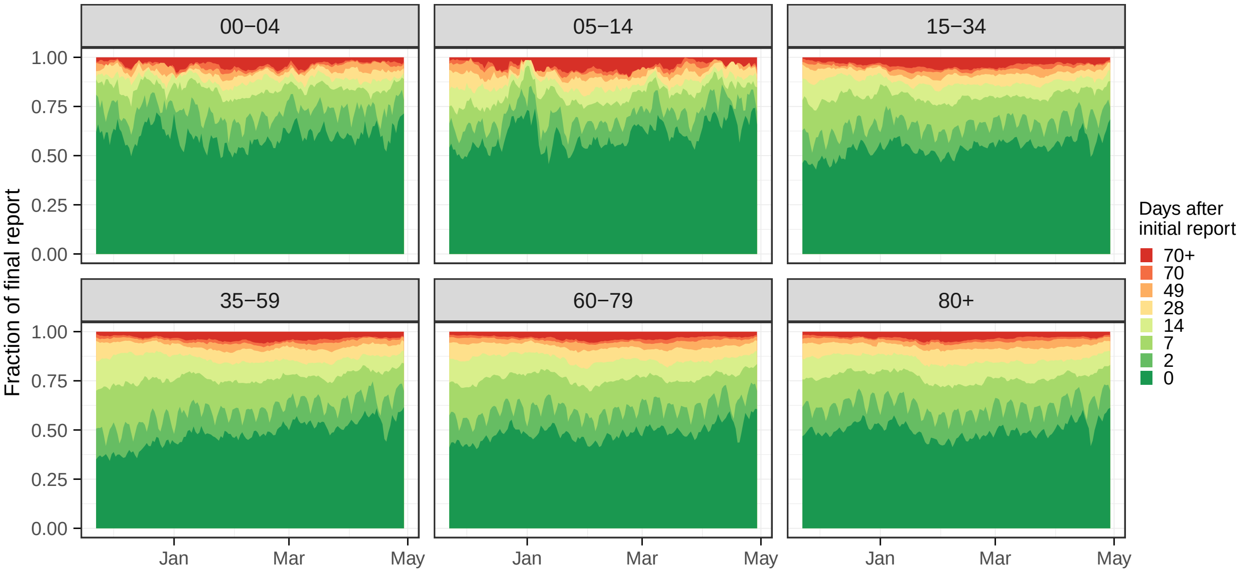

Fig A2: **Temporal development of the reporting completeness in different age groups.** Shown is the reported fraction of the final 7-day hospitalization incidences 0 to 70 days after the respective reference date.

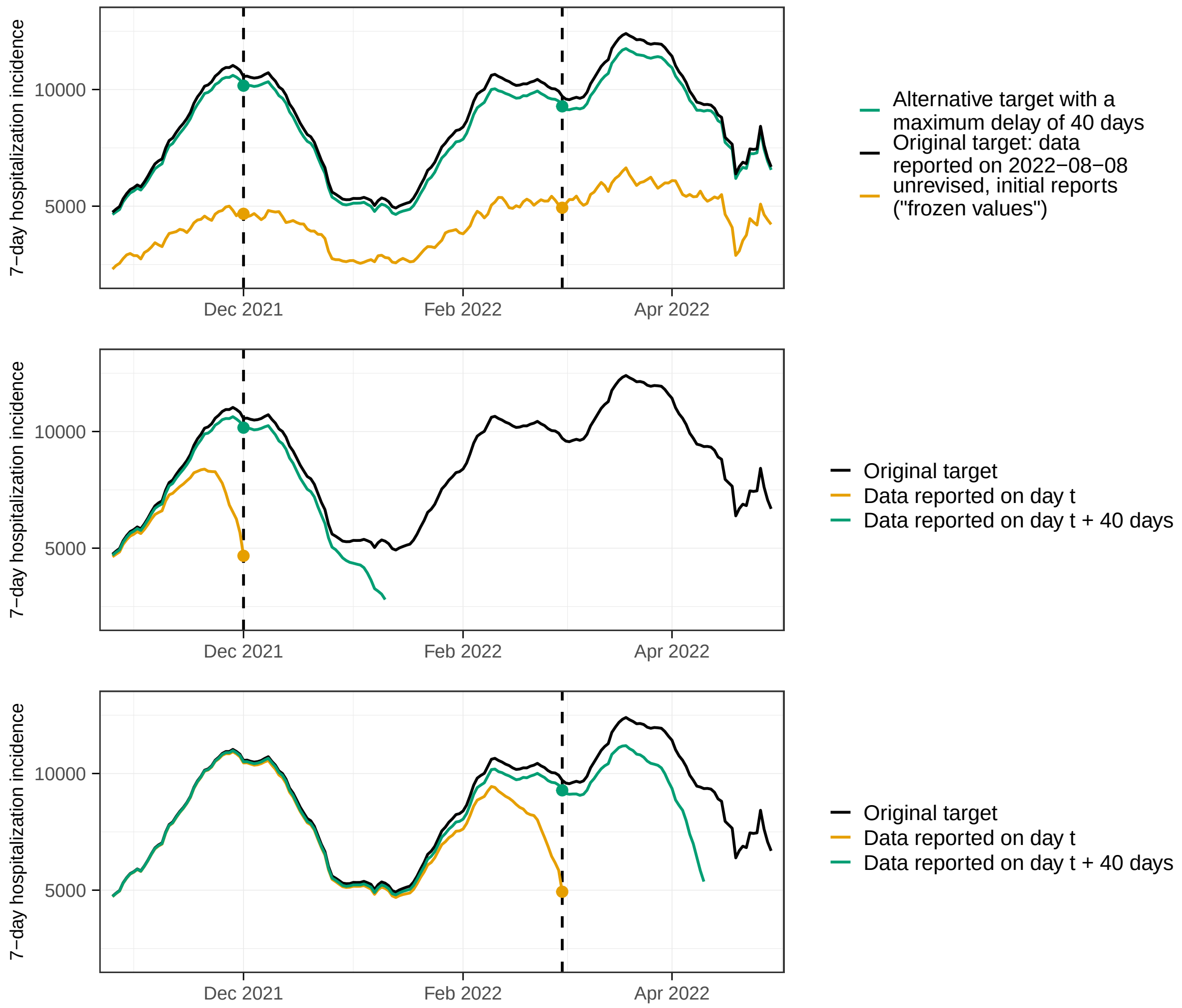

Fig A3: **Schematic illustration of the alternative target with a maximum delay of 40 days.** This target (green) was defined in Section 3.7 and is compared to the original target (black) and the frozen values (yellow). In the new target, hospitalizations are only counted if they happen within 40 days after the case report, while in the original target, there was no upper limit on delays. The top panel shows the final version of each time series. The two bottom panels show how they arise from real-time data.

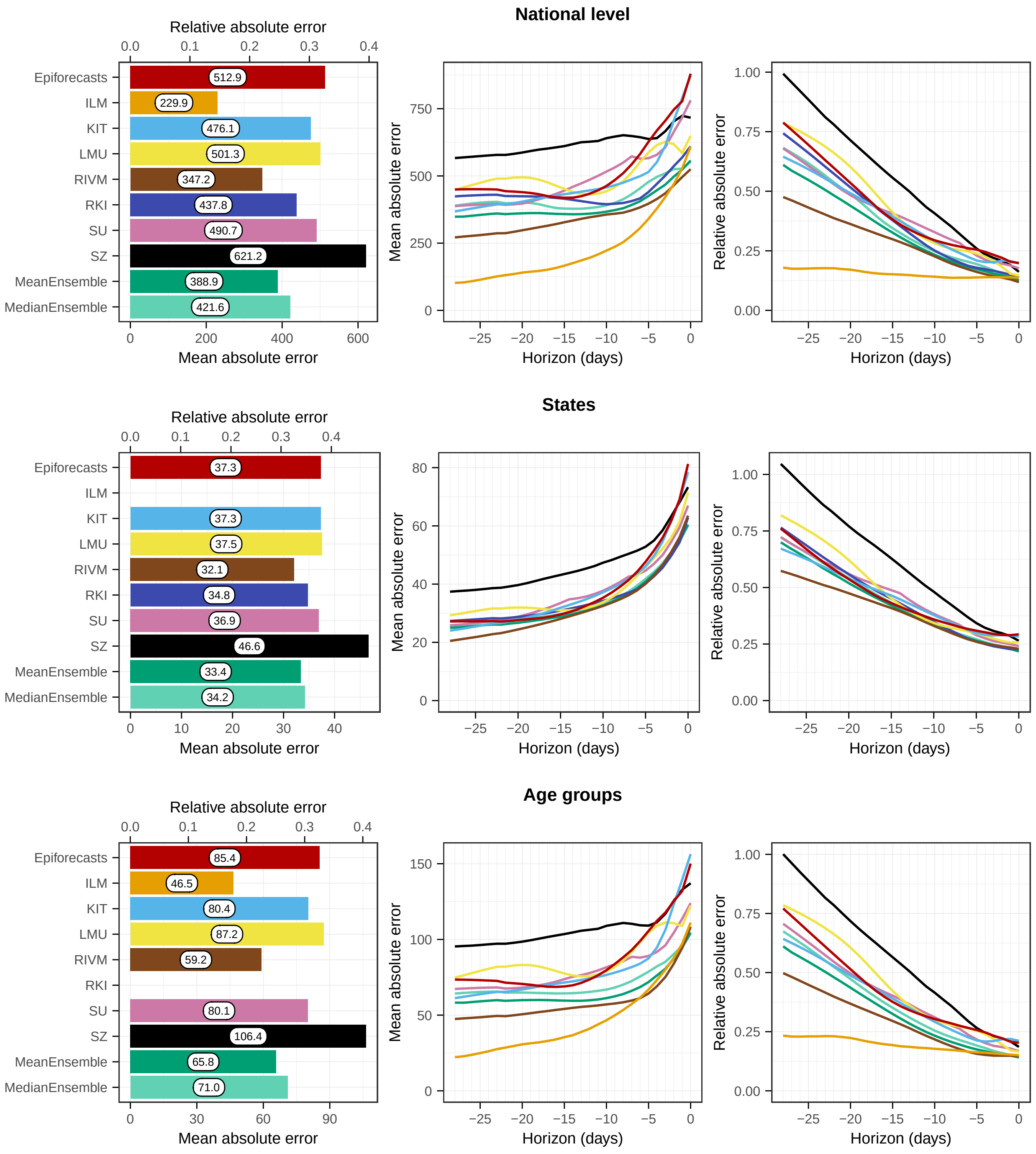

Fig A4: **Performance of the point predictions (predictive medians).** Shown are the mean absolute errors for the national level (top) and averaged across states (middle) and age groups (bottom). The first panel in each row displays the average across all horizons (on the absolute and relative scales). The second and third panels in each row show the mean absolute error and the relative absolute error, respectively, stratified by horizon.

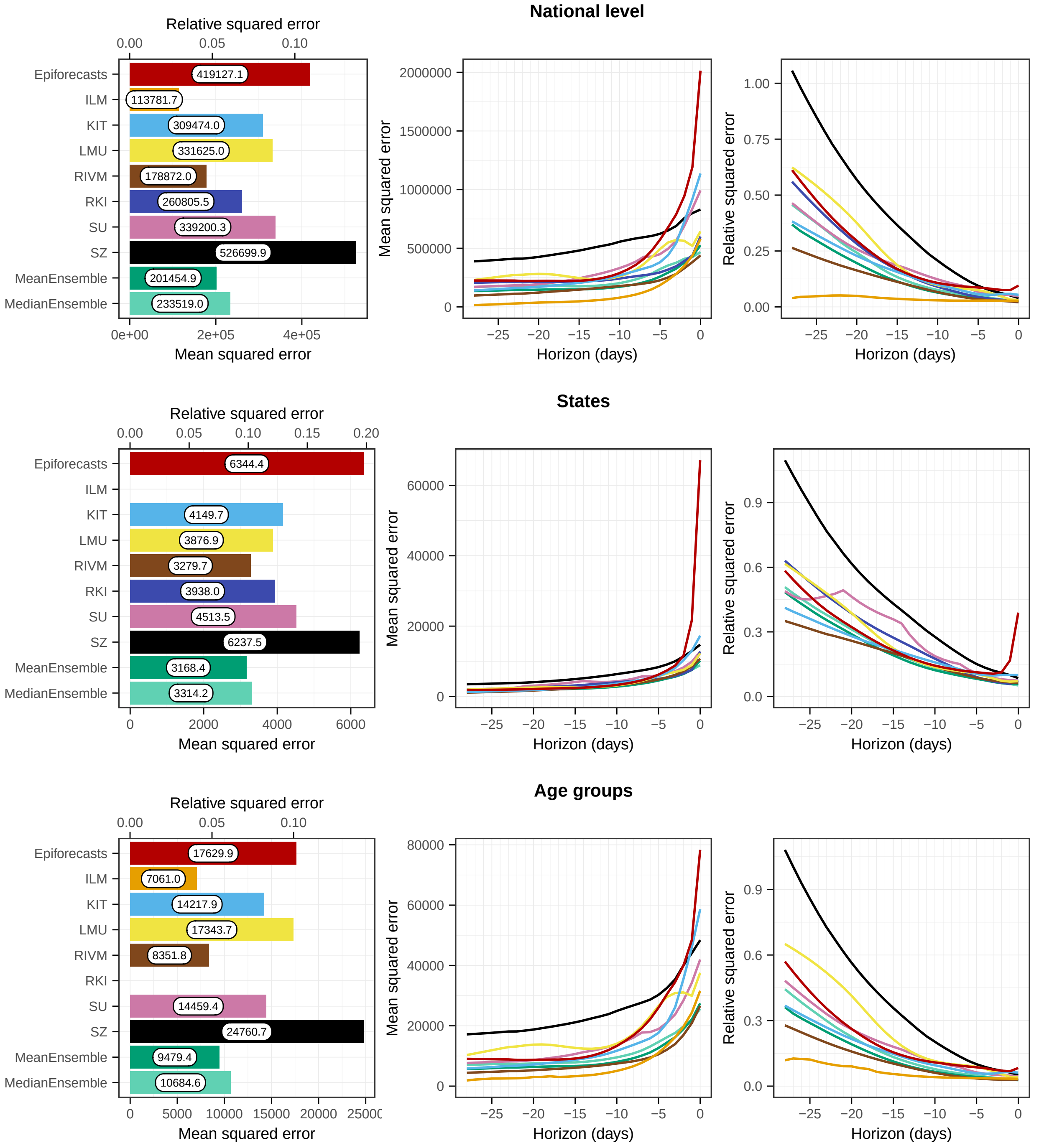

Fig A5: **Performance of the point predictions (expected values).** Shown are the mean squared errors for the national level (top) and averaged across states (middle) and age groups (bottom). The first panel in each row displays the average across all horizons (on the absolute and relative scales). The second and third panels in each row show the MSE and the relative MSE, respectively, stratified by horizon.

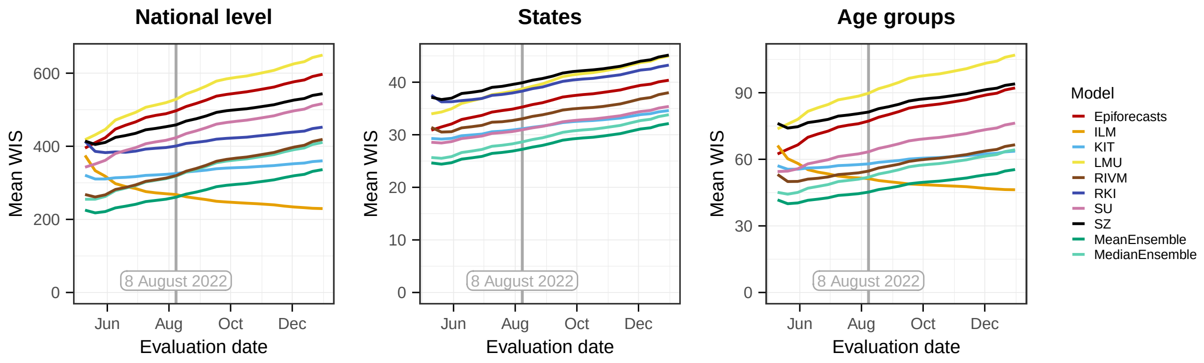

Fig A6: **Performance on short horizons based on the chosen "final" data.** Shown is the mean WIS across horizons from 0-7 days computed using different data versions as the "final" version. The version prespecified in the study protocol is 8 August 2022, marked by a vertical line.

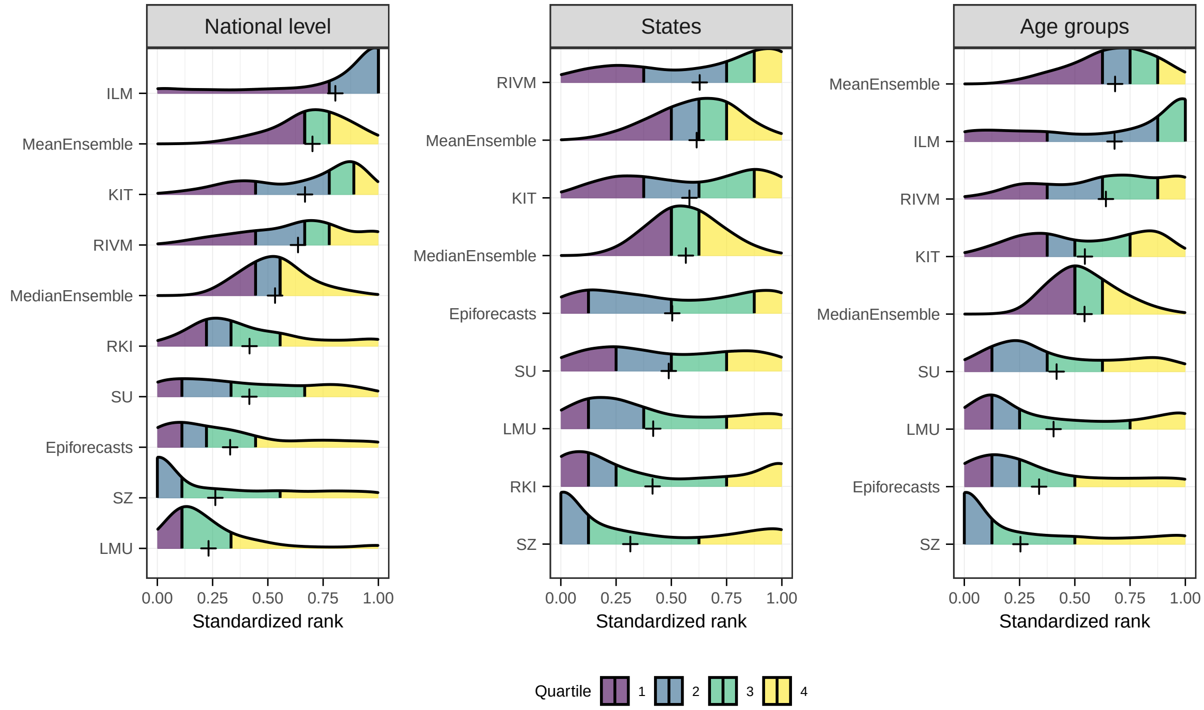

Fig A7: **Model rank distributions.** Distribution of each model's standardized rank for each nowcast-observation pair (see [1] for details on the definition) The models are ordered by the mean standardized rank, which is indicated by a plus sign.

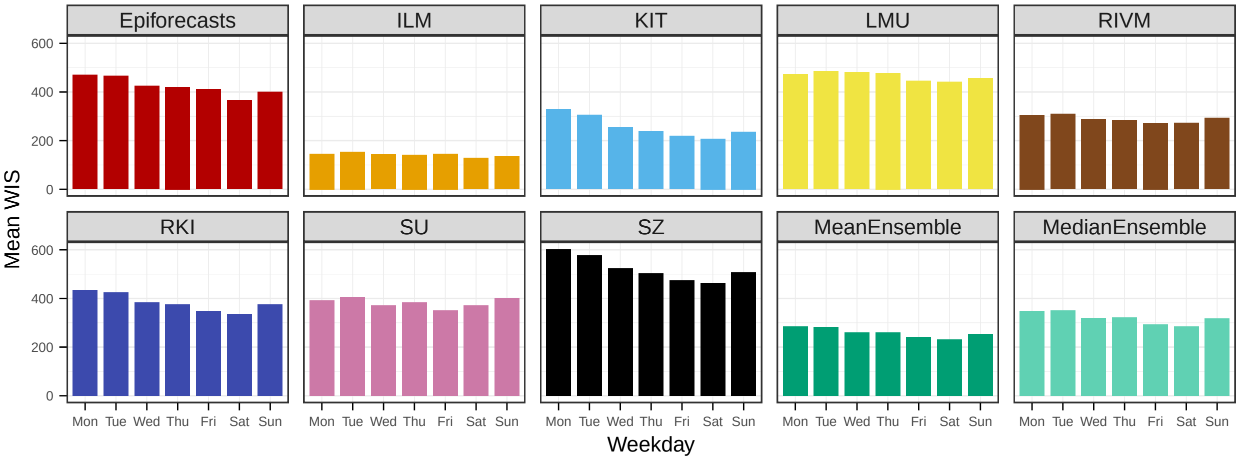

Fig A8: **Impact of weekday effects on the scores.** Mean WIS of same-day nowcasts (with a horizon of 0 days) averaged by weekday.

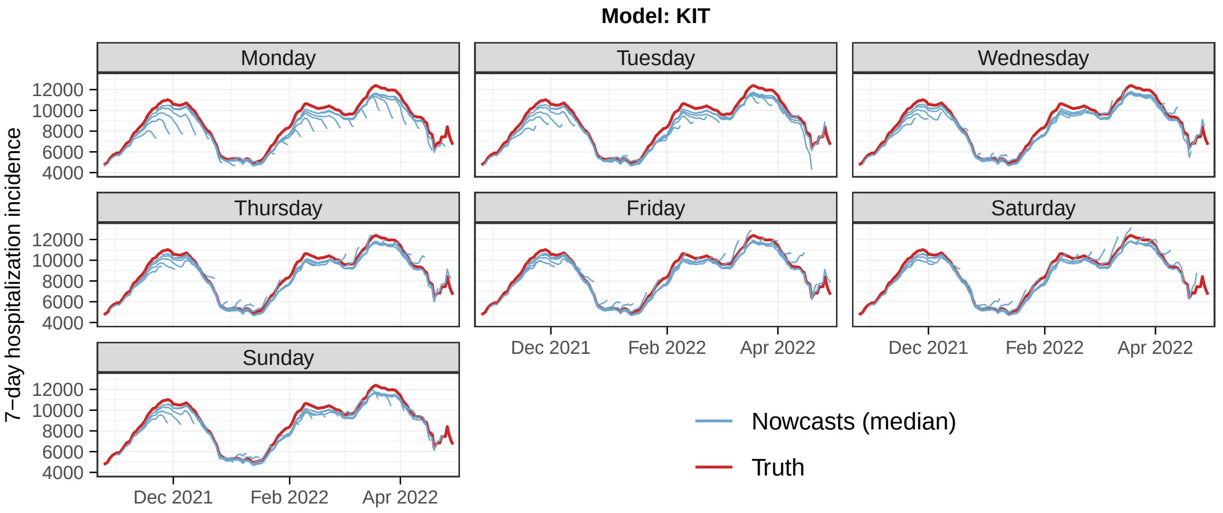

Fig A9: **Nowcasts of the KIT model issued on different weekdays (0 to 28 days back).** Nowcasts issued on Mondays show strong downward biases, while on Saturdays both under- and overprediction occur.

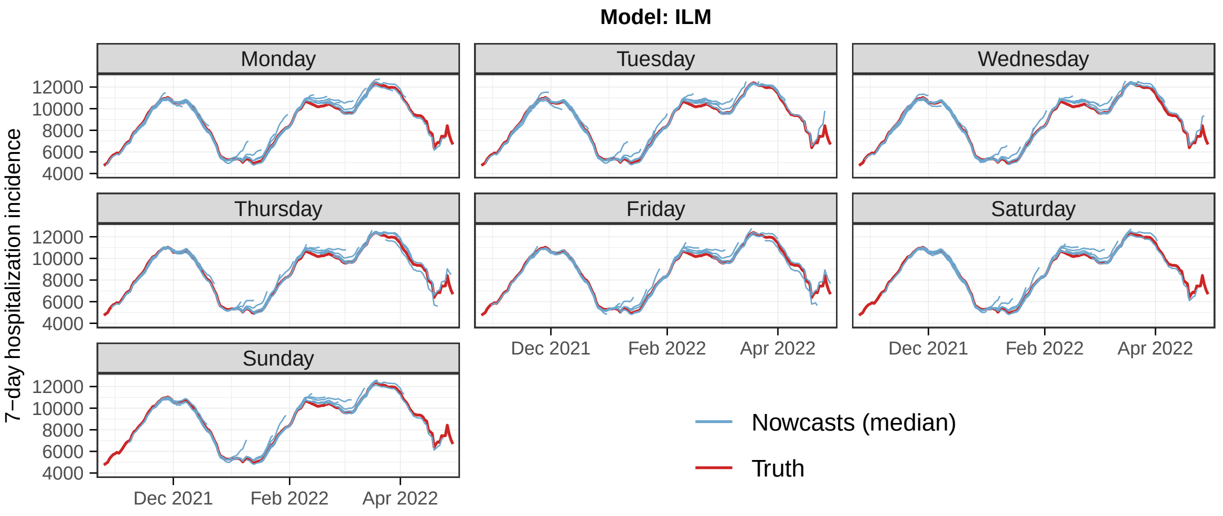

Fig A10: **Nowcasts of the ILM model issued on different weekdays (0 to 28 days back).** No clear weekday patterns can be discerned.

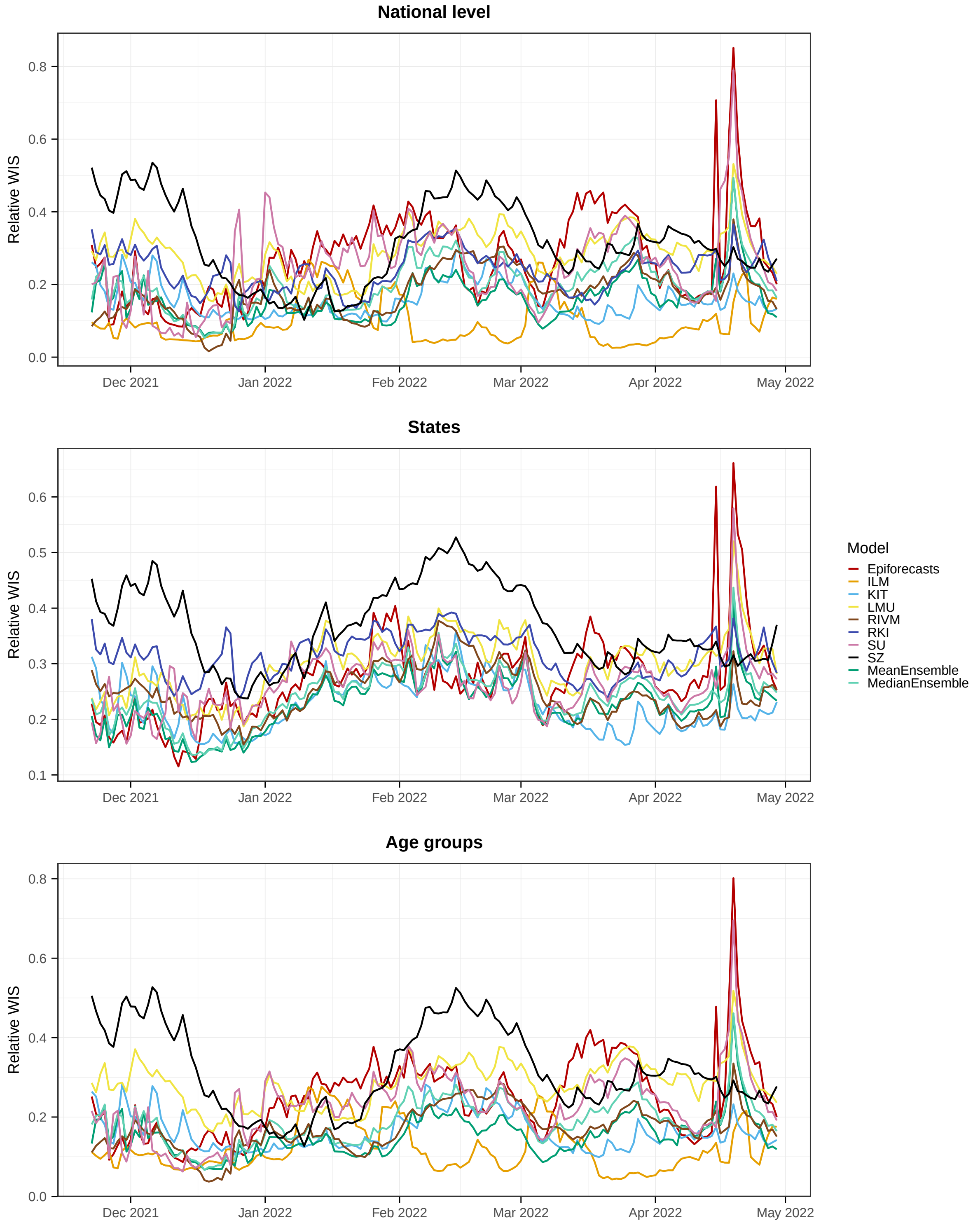

Fig A11: **Performance over time.** Relative mean WIS across all horizons by nowcast date and stratification level. Top: national level; middle: across states; bottom: across age groups.

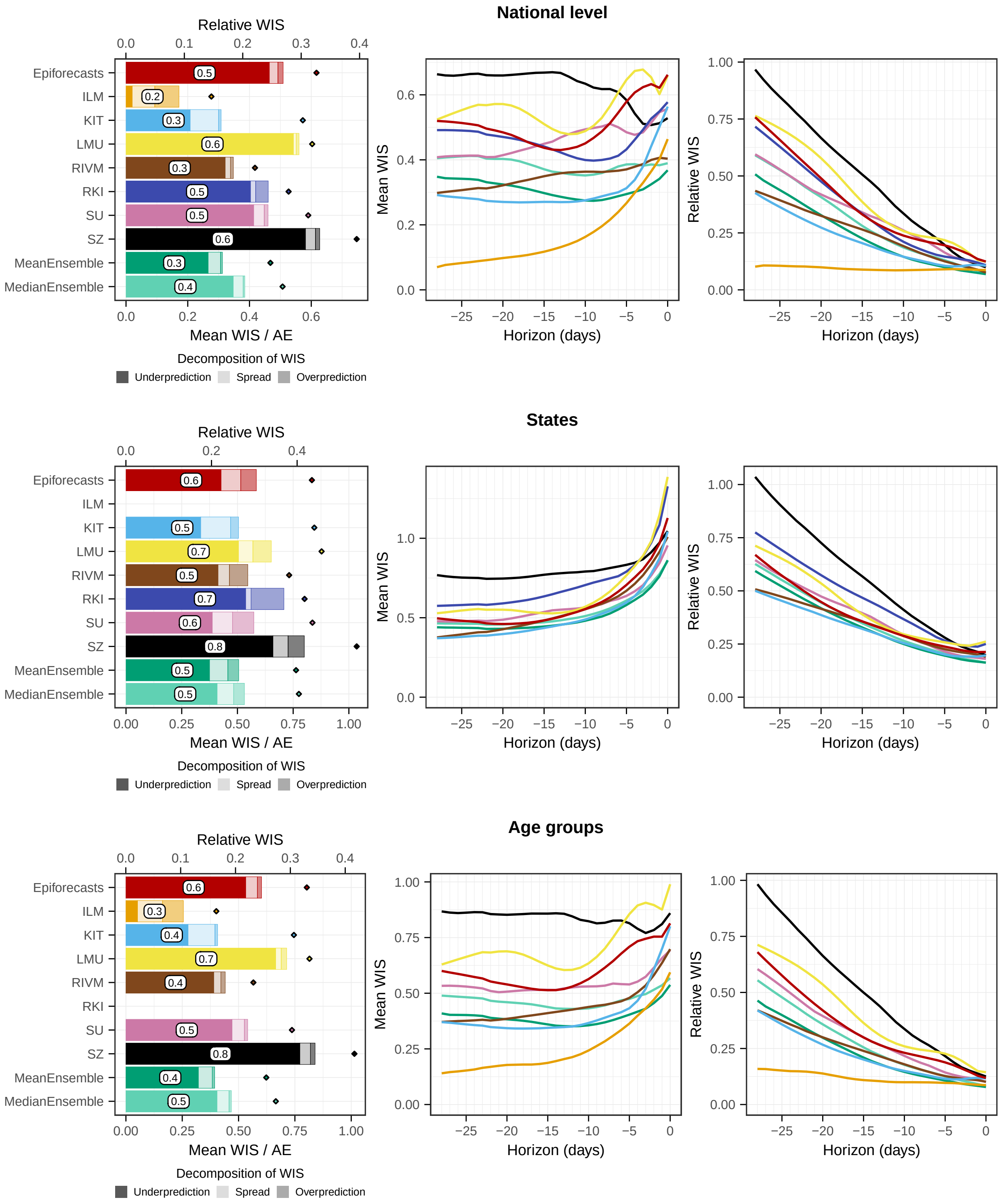

Fig A12: **Scores computed after standardization by population.** Shown are the mean WIS and AE as in Figure 7 but for hospitalizations by 100,000 inhabitants. This gives similar weight to all states irrespective of their population size.

B Deviations from study protocol and completeness of nowcasts

As we have deviated in some minor parts from the study protocol, we provide a list of these adjustments.

Table A1: Deviations from the study protocol.

Description	Reasoning
Inclusion of weekends into the study period.	Initially, weekends were excluded from the study period as we expected submission to require some human intervention at least initially. As all teams completely automated their procedures very quickly, we were able to update models on weekends and include them in the evaluation.
Inclusion of the Christmas period into the study period.	In the study protocol, the Christmas period was excluded from the study period as (i) we expected irregular reporting behavior and (ii) we did not want to oblige modelers to perform any manual steps for submission during this period. However, as there were no unusual patterns and all submissions were completely automated, we decided to include this period. We note that, contrary to our expectations, the Easter period showed some unusual reporting patterns. We nonetheless kept it in the evaluation as removing periods for which nowcasts were expected to work normally but did not would unduly embellish our results.
Omission of the retrospective study period from 1 July 2021 to 19 November 2021.	We initially planned to include a retrospective study period in order to contrast the performance of methods in retrospect and in real time. However, as only two teams provided retrospective nowcast before the start of the study period, we chose to omit this aspect. Instead, we chose to include an analysis of four revised versions of contributed models applied retrospectively to the period 22 November to 29 April.
Omission of nowcast targets for which even including fill-in nowcasts results could not be obtained from all methods.	For a very small set of targets we were unable to obtain submissions from all models, even including fill-in nowcasts. As these represented a negligible fraction of all targets (0.3%) we pragmatically chose to omit these from the main analysis in order to achieve a balanced data set.
Omission of interval coverage results at the 80% level.	As interval coverage results at the 80% level provided no additional insights and led to overly full figures we chose to omit them.
Definition of naïve baseline model.	At the time of writing the protocol we had decided that a naïve baseline model should be included, but it was unclear how it should be defined. The `FrozenBaseline` used in the main analysis was only defined during the work on the manuscript.
Tightening of ensemble inclusion criteria.	During the study period, we realized that one model (`SZ`) occasionally issued nowcast values below the already known values (which in almost all cases only get corrected upwards). This is due to a smoothing step that is included in the procedure. We decided to exclude these from the ensemble. Specifically, submissions were excluded from the ensemble whenever the median or mean nowcast was below the already known value.

Table A2: Missingness of real-time submissions by the participating teams. Apart from the targets listed in Table A3, all of these could be imputed with fill-in nowcasts.

Model	Dates without submission
`Epiforecasts`	25–26 Jan 2022
`ILM`	27–28 Nov 2021, 24, 26, 30 Dec 2021, 16 Jan 2022, 8–20 April 2022
`RIVM`	8 Dec 2021, 23 Apr 2022
`RKI`	8 Dec 2021; all 0 and -1 day nowcasts
`SU`	27–28 Nov 2021, 5 Dec 2022

Table A3: Nowcast targets for which no complete sets of submissions could be obtained. These amount to 394 nowcast targets among the 109,968 considered in total.

Dates	Excluded targets	Reason
22 November 2021	Horizons -1 and 0 days, all strata	On the first day of our study several models provided only nowcasts from -2 days backward.
22–24 November 2021	Horizons -23 to -28 days, all strata.	The `SZ` model initially only provided nowcasts three weeks back.
31 Jan, 1 Feb 2022	State of Hamburg, all horizons.	Nowcasts from `Epinowcasts` model not available due to convergence issues.

Table A4: Other decisions in response to unexpected difficulties.

Description	Reasoning
Imputation of 0.1-quantiles from `Epiforecasts` model via an interpolation/normal approximation.	For several months the `Epiforecasts` model did not provide 0.1 quantiles, which was only noticed towards the end of the study period. in order to be able to evaluate the WIS without having to rerun the model for all concerned dates, we imputed the 0.1-quantile by interpolating between the 0.025 and the 0.25 quantile based on a normal approximation (which implies that the 0.1 quantile is almost exactly halfway between the 0.025 and 0.25 quantiles).
`LMU` nowcasts for Saarland and Bremen were replaced by nowcasts from the retrospectively revised model version discussed in Section 3.6.	In their real-time submissions, the `LMU` team only reported point nowcasts for the states of Saarland and Bremen, which are considerably smaller than the other states (1M and 700k inhabitants, respectively). To be able to nonetheless evaluate the WIS and keep these two states in the overall evaluation, we used the revised nowcasts as discussed in Section 3.6 as these contained all quantiles for these states. We consider this defensible as the role of these two states in the overall evaluation under WIS is very small (the WIS typically scales with the order of magnitude of the target).
Filling in some missing entries of `ILM` with nowcasts from the updated model.	Nowcasts from 22-26 and 29 November 2021 were missing entries for horizons -28, -1, and 0 days. To fill these in, we used the revised nowcasts as discussed in Section 3.6.
Removal of a small number of obviously erroneous nowcasts for the `RKI` model.	In a handful of instances, the `RKI` model submitted obviously erroneous nowcasts for the 0-day horizon. These stated values of more than 1 billion hospitalizations. We replaced these with the respective -1 day nowcasts.

C Repositories of participating teams

- Epiforecasts: `https://epiforecasts.io/eval-germany-sp-nowcasting/`
- ILM: `https://github.com/Stochastik-TU-Ilmenau/ILM-prop`
- KIT: `https://github.com/KITmetricslab/hospitalization-nowcast-hub/tree/main/code/baseline`
- LMU: `https://github.com/MaxWeigert/Nowcasting_covid19_hospitalizations`
- RIVM: `https://github.com/kassteele/Nowcast-hub`
- SU: `https://github.com/FelixGuenther/hospitalization-nowcast-hub_SU-public`

D Sensitivity analysis via pairwise comparisons

D.1 Motivation and procedure

In some instances, teams failed to submit nowcasts in time and had to fill them in post hoc. Allowing them to do so may seem lenient as, in principle, teams could use additional information, thus unfairly improving their nowcasts. As specified in our protocol, we thus perform a sensitivity analysis based purely on nowcasts submitted on time of the respective day.

As each team failed to submit nowcasts on different days this leads to a setting where methods need to be compared based on incongruent sets of nowcasting tasks. In this setting, the relative WIS could still be evaluated for each method by considering only the subset of targets treated by the respective method. This, however, ignores that improving upon the naïve baseline is easier for certain locations, age groups, and time periods than others. To handle this difficulty, we use the *pairwise comparison* approach suggested in [1]. It is based on the assumption that achieving good nowcast performance *relative to all other considered methods* is similarly difficult across locations, age groups, and time periods. Considering a set of N models (including the baseline model), the relative WIS corrected for missing submissions is determined as follows:

1. In the first step for each pair of models i, j we compute the ratio
$$\theta_{ij} = \frac{\text{mean WIS achieved by model } i}{\text{mean WIS achieved by model } j}.$$
2. For each model i we then compute the geometric average of the ratios θ_{ij} achieved in the comparisons to all other models
$$\theta_i = \left(\prod_{i=1}^{N} \theta_{ij}\right)^{1/N}.$$
3. Lastly, we re-scale the θ_i to the one achieved by the baseline model to obtain the relative WIS:
$$\text{adjusted relative WIS of model } i = \frac{\theta_i}{\theta_{\text{BL}}},$$
where θ_{BL} refers to the baseline model (`FrozenBaseline`).

If all models submitted all required nowcasts it is straightforward to show that this boils down to the regular relative WIS as defined in Section 2.5. If some submissions are missing for certain models, the procedure will adjust the relative WIS to how well other models fared on the respective subset of addressed targets.

D.2 Results

Table A5 compares the relative WIS computed using fill-in nowcasts as in the main analysis and the pairwise comparison approach. The differences are very modest, meaning that the missingness of nowcasts does not substantially affect the results. This is not surprising, given the low number of missing submissions.

Table A5: Comparison of relative WIS values obtained using retrospective fill-in nowcasts and the pairwise comparison approach from [1] (PC).

	National level		States		Age groups	
	PC	fill-in	PC	fill-in	PC	fill-in
`Epiforecasts`	0.2679	0.2690	0.2686	0.2686	0.2465	0.2472
`FrozenBaseline`	1.0000	1.0000	1.0000	1.0000	1.0000	1.0000
`ILM`	0.0907	0.0907	–[1]	–[1]	0.1124	0.1117
`KIT`	0.1627	0.1628	0.2227	0.2232	0.1676	0.1677
`LMU`	0.2961	0.2962	0.2993	0.2988	0.2925	0.2926
`MeanEnsemble`	0.1643	0.1651	0.2254	0.2254	0.1574	0.1581
`MedianEnsemble`	0.2034	0.2034	0.2414	0.2415	0.1919	0.1920
`RIVM`	0.1845	0.1838	0.2471	0.2473	0.1767	0.1764
`RKI`	–[2]	0.2435	–[2]	0.3120	–[1]	–[1]
`SU`	0.2427	0.2432	0.2590	0.2585	0.2265	0.2268
`SZ`	0.3314	0.3317	0.3703	0.3709	0.3330	0.3332

[1] No nowcasts submitted for this target.

[2] WIS could only be evaluated for fill-in nowcasts as real-time submissions did not contain all required quantiles.

E Documentation of the `KIT` model

As the `KIT` model was conceived as a conceptually simple (though not naïve) reference model for the current study we provide a brief documentation of its methodology.

Notation Denote by $X_{t,d}, d = 0, \dots, D$ the number of hospitalizations for reference date t which appear in the data set at day $t + d$ and by

$$X_{t,\leq d} = \sum_{i=0}^{d} X_{t,i}$$

the number of hospitalizations reported for reference date t up to day $t + d$. Moreover, denote by

$$X_t = X_{t,\leq D} = \sum_{i=0}^{D} X_{t,i}$$

the total number of reported hospitalizations for t, where D denotes an assumed maximum possible delay. In the following, we denote by X_t, etc. a random variable and by x_t the corresponding observation.

The observed $x_{t,d}$ as available at a given time point t^* can be arranged into the so-called *reporting triangle*, see Table A6.

As we will focus on seven-day hospitalization incidences we moreover need to consider rolling sums over windows of length W (usually $W = 7$)

$$Y_t = \sum_{w=0}^{W-1} X_{t-w}.$$

Goal Our aim is to estimate or *nowcast* Y_t based on the information available at time $t^* \geq t$. We do not take into account any information other than data on hospitalizations and their reporting delays, meaning that we model

$$Y_t \mid X_{s,d} : s + d \leq t^*, d \geq 0.$$

Table A6: Illustration of the reporting triangle for time t^* and $D = 5$. Quantities known at time t are shown in black, yet unknown quantities are shown in gray.

day	$d = 0$	$d = 1$	$d = 2$	$d = 3$	$d = 4$	$d = 5$	total
1	$x_{1,0}$	$x_{1,1}$	$x_{1,2}$	$x_{1,3}$	$x_{1,4}$	$x_{1,5}$	x_1
2	$x_{2,0}$	$x_{2,1}$	$x_{2,2}$	$x_{2,3}$	$x_{2,4}$	$x_{2,5}$	x_2
$\vdots$							
$t^* - 5$	$x_{t^*-5,0}$	$x_{t^*-5,1}$	$x_{t^*-5,2}$	$x_{t^*-5,3}$	$x_{t^*-5,4}$	$x_{t^*-5,5}$	x_{t^*-5}
$t^* - 4$	$x_{t^*-4,0}$	$x_{t^*-4,1}$	$x_{t^*-4,2}$	$x_{t^*-4,3}$	$x_{t^*-4,4}$	$x_{t^*-4,4}$	x_{t^*-4}
$t^* - 3$	$x_{t^*-3,0}$	$x_{t^*-3,1}$	$x_{t^*-3,2}$	$x_{t^*-3,3}$	$x_{t^*-3,4}$	$x_{t^*-3,5}$	x_{t^*-3}
$t^* - 2$	$x_{t^*-2,0}$	$x_{t^*-2,1}$	$x_{t^*-2,2}$	$x_{t^*-2,3}$	$x_{t^*-2,4}$	$x_{t^*-2,5}$	x_{t^*-2}
$t^* - 1$	$x_{t^*-1,0}$	$x_{t^*-1,1}$	$x_{t^*-1,2}$	$x_{t^*-1,3}$	$x_{t^*-1,4}$	$x_{t^*-1,5}$	x_{t^*-1}
t^*	$x_{t^*,0}$	$x_{t^*,1}$	$x_{t^*,2}$	$x_{t^*,3}$	$x_{t^*,4}$	$x_{t^*,5}$	x_{t^*}

Point nowcast The following describes a simple heuristic to obtain a point prediction of Y_t based on information available at time t^*.

We start by imputing

$$x_{t^*,1} = x_{t^*,0} \times \frac{\sum_{i=1}^{t^*-1} x_{t^*-i,1}}{\sum_{i=1}^{t^*-1} x_{t^*-i,0}},$$

i.e. use a simple multiplication factor computed from the complete rows of our data set. Next, we compute

$$x_{t^*,2} = x_{t^*,\leq 1} \times \frac{\sum_{i=1}^{t^*-1} x_{t^*-i,2}}{\sum_{i=1}^{t^*-1} x_{t^*-i,\leq 1}},$$

where in the computation of

$$x_{t^*-i,\leq 1} = x_{t^*-i,\leq 0} + x_{t^*-i,1}$$

we just treat the $x_{t^*-i,1}$ imputed in the first step as if it was a known value. The same can be done for

$$x_{t^*-1,2} = x_{t^*-1,\leq 1} \times \frac{\sum_{i=1}^{t^*-1} x_{t^*-i,2}}{\sum_{i=1}^{t^*-1} x_{t^*-i,\leq 1}}.$$

We repeat this same procedure to fill in the missing values of the reporting triangle step by step, moving from the left to the right and the bottom to the top.

This is equivalent to the following slightly more formal formulation: We denote by π_d the probability that a hospitalization with reference date t appears in the data on day $t + d$ and by

$$\pi_{\leq d} = \sum_{i=0}^{d} \pi_i$$

the probability that such a hospitalization appears in the data no later than $t + d$. We introduce

$$\theta_d = \frac{\pi_d}{\pi_{\leq d-1}},$$

which allows us to formulate the recursion

$$\pi_{\leq d} = (1 + \theta_d)\pi_{\leq d-1}.$$

To estimate the θ_d for $d = 1, \ldots, D < t$ based on quantities available at time t^* we use

$$\hat{\theta}_d(t^*) = \frac{\sum_{j=d}^{J} X_{t^*-j,d}}{\sum_{j=d}^{J} X_{t^*-j,\leq d-1}},$$

where J is the number of past observations to include in the estimation (in practice it is often helpful to use only a recent subset rather than the entire available history). Note that we treat this estimate as a function of t^* as it may change over time. Estimates of the probabilities $\pi_{\leq d}$ can then be obtained as

$$\hat{\pi}_{\leq d}(t^*) = (1 + \hat{\theta}_d)\hat{\pi}_{\leq d-1}.$$

These can subsequently serve to estimate the total number X_t of hospitalizations with reference date t based on the $X_{t,\leq t^*-t}$ hospitalizations already reported by time t^*:

$$\hat{X}_t(t^*) = \frac{X_{t,\leq t^*-t}}{\hat{\pi}_{\leq t^*-t}(t^*)}.$$

We can also compute the estimates for the respective number of hospitalizations reported with a given delay $d > t^* - t$, which is given by

$$\hat{X}_{t,d}(t^*) = \hat{\pi}_d(t^*)\hat{X}_t(t^*).$$

In the last step we move to the rolling sum Y_t, which we estimate as

$$\hat{Y}_t(t^*) = \sum_{w=0}^{W-1} \hat{X}_{t-w}(t^*).$$

Uncertainty quantification Our general idea to quantify the nowcast uncertainty for $\hat{Y}_t(t^*)$ is to generate point nowcasts $\hat{Y}_{t-1}(t^*-1), \hat{Y}_{t-2}(t^*-2), \ldots, \hat{Y}_{t-K}(t^*-K)$ for $K > D$ past time points, each based on the information available at the respective time point. These could then be compared to the corresponding observations $Y_{t^*-1}, \ldots, Y_{t^*-K}$, and nowcast dispersion could be based on a simple parametric model. However, two aspects need to be taken into account:

- The information available at t^*, on which the nowcast $\hat{Y}_t(t^*)$ is based, already implies a lower bound for Y_t, namely the hospitalizations which have already been observed. Only the hospitalizations for reference date t which will be reported after t^* need to be modeled probabilistically. We thus introduce the decomposition

  $$Y_t = Y_{t,\leq t^*-t} + Y_{t,>t^*-t}.$$

  Here,

  $$Y_{t,\leq t^*-t} = \sum_{w=0}^{W-1} \sum_{d=0}^{D} X_{t-w,d} \times \mathbb{I}(-w + d \leq t^* - t)$$

  are those already observed by t^* (i.e., the lower bound) and

  $$Y_{t,>t^*-t} = \sum_{w=0}^{W-1} \sum_{d=0}^{D} X_{t-w,d} \times \mathbb{I}(-w + d > t^* - t)$$

  are those yet to be observed. We only need to quantify the uncertainty about the latter.

- At time t^*, the realizations of $Y_{t,>t^*-t}$ are only available for $t \leq t^* - D$. If we only want to use complete observations we would need to discard a lot of recent information.

We therefore construct a set of observations $Z_{t-j,>t^*-t}, j = 1, \ldots, K$ and corresponding point predictions $\hat{Z}_{t-j,>t^*-t}, (t^* - j)$ as follows:

- For $j = D, \ldots, K$ we can simply set

  $$Z_{t-j,>t^*-t} = Y_{t-j,>t^*-t}$$

  and point predictions $\hat{Z}_{t-j,>t^*-t}(t^* - j) = \hat{Y}_{t-j,>t^*-t}(t^* - j)$ as all relevant information are already available at t^*.

- For $j = 1, \dots, D-1$ we use partial observations

$$Z_{t-j,>t^*-t} = \sum_{w=0}^{W-1} \sum_{d=0}^{D} X_{t-j-w,d} \times \mathbb{I}(\underbrace{t-j-w+d \leq t^*}_{\text{"already observed at } t^*\text{"}}),$$
$$= Y_{t-j,>t^*-t} - Y_{t-j,>t^*-t+j}$$

which are restricted to hospitalizations already reported by time t^*, so that $Z_{t-j,>t^*-t}$ can be evaluated. The corresponding point nowcasts are given by

$$\hat{Z}_{t-j,>t^*-t} = \sum_{w=0}^{W-1} \sum_{d=0}^{D} \hat{X}_{t-j-w,d} \times \mathbb{I}(\underbrace{t-j-w+d \leq t^*}_{\text{"already observed at } t^*\text{"}}).$$

We then pragmatically assume that

$$Z_{t-j} \mid \hat{Z}_{t-j}(t^* - j) \quad \sim \quad \text{NegBin}(\text{mean} = \hat{Z}_{t-j}(t^* - j), \text{disp} = \psi_{t^*-t}),$$

where we parameterize the negative binomial distribution via its mean and the dispersion (size) parameter ψ_{t^*-t}. Note that the dispersion parameter depends on how far back into the past we nowcast (i.e., how much information has already accumulated between $t-j$ and $t^* - j$). The parameters $\psi_0, \dots, \psi_D$ are then estimated via maximum likelihood. To avoid issues with zero expectations we add 0.1 to the expected values when feeding them into the maximum likelihood procedure.

The predictive distributions for Y_t are then set to $\text{NegBin}(\text{mean} = \hat{Y}_{t,>t^*-t}(t^*), \text{size} = \psi_{t^*-t})$, shifted by $Y_{t,\leq t^*-t}$. As a motivation for the use of partial observations in the estimation of the overdispersion parameters, we note that if

$$A \sim \text{NegBin}(\text{mean} = \hat{A}, \text{disp} = \psi)$$

and

$$B \mid A \sim \text{Bin}(A, \pi)$$

one gets

$$B \sim \text{NegBin}(\text{mean} = \pi\hat{A}, \text{disp} = \psi).$$

The negative binomial distribution with a given dispersion parameter is thus closed to binomial subsampling, with only the expectation, but not the size parameter changing. It is therefore defendable to assume the same size parameter for the constructed partial observations $Z_{t-j,>t^*-t}$ and the actual $Y_{t-j,>t^*-t}$ which we would use if they were already available.

Parameter choices To apply the suggested method, the numbers J and K of past observations are used to estimate the nowcast mean and dispersion parameters. Here one needs to strike a balance between a sufficient amount and recency of training data. We set both J and K to 60 days without further assessing the impact on nowcast quality. The maximum delay of D was set to 40 days.

References

[1] E. Y. Cramer, E. L. Ray, V. K. Lopez, J. Bracher, *et al.*, "Evaluation of individual and ensemble probabilistic forecasts of COVID-19 mortality in the United States," *Proceedings of the National Academy of Sciences*, vol. 119, no. 15, e2113561119, 2022.
