# Supplementary material for: Collaborative nowcasting of COVID-19 hospitalization incidences in Germany
Source: PLoS Comput Biol. 2023 Aug 11;19(8):e1011394. doi: 10.1371/journal.pcbi.1011394 (PMC10446237; doi:10.1371/journal.pcbi.1011394)
Supplement: Attachment — Submitted filename: report.pdf [file pcbi.1011394.s002.pdf]

Referee report

PCOMPBIOL-D-23-00600: Collaborative nowcasting of COVID-19 hospitalization incidences in Germany

25 May 2023

This paper examines the results of a collaborative, preregistered, nowcasting experiment on German hospitalizations during a 6 month period of the COVID-19 pandemic. The analysis is thorough and carefully described, and the results are of general interest to the community.

Minor comments

1. In section 2.1, the description of the target and reference date is rather complicated. I suggest adding a figure describing how to understand what this target is and how it relates to some of the issues discussed later in the manuscript.
2. Section 2.4. On the Ensembling, what happens if the resulting aggregated quantiles cross? Perhaps this never happened given the wide set of intervals, but it's worth thinking about, and mentioning as a potential issue. (Also, potentially for the individual models if they aren't derived from distributional forecasts). I believe one can show that sorting the quantiles is guaranteed to improve WIS, e.g if a forecast was `(2, 1, 5, 7, 9, 15, 20)` for the quantiles on line 150, simply sorting `(1, 2, 5, 7, 9, 15, 20)` is better.
3. In addition to the Relative Arithmetic Mean WIS described in section 2.5, I'd suggest reporting the Relative Geometric Mean WIS. There are a few reasons for this suggestion. (1) It's equivalent to the statistic in the paper, but using `mean(log(WIS))` rather than WIS which is generally right skewed. (2) It has the simultaneous interpretation as the geometric Mean of `WIS(m) / WIS(b)`, so that it can be seen as normalizing the error for a particular nowcast by degree-of-difficulty as given by the baseline. This latter interpretation also allows missing forecasts to be dropped without arbitrarily helping/hurting the overall score. (Now on looking at the Supplement, this is just a transformation of the pairwise comparison in C.1, right?)
4. Section 3.3. It might be worth creating a figure to examine Relative WIS over time, rather than only over the whole period. It could go in the Supplement.
5. Still section 3.3 (lines 254 and 262). It would be helpful here to have a sentence in each case that explains (or at least conjectures) *why* KIT has these deficiencies. In the second case, it seems plausibly due to the Max Delay (examined elsewhere), but it would be nice to at least point to that possibility here.
6. The finding described on 292–93 strikes me as important. Maybe worth highlighting elsewhere as well? Seems like an important lesson for improving nowcasting models.
7. Section 3.7 I don't quite understand the "rolling" approach as described. It would be good to do 1 or more of (a) give a concrete example, (b) explain mathematically, or (c) create a figure illustrating how to create this target.
8. Given the success of `ILM` it would really help to have more detail about how it works. There's no reference or inclusion in Appendix D. So it reads as a blackbox that seems to do well. Is there at least a README or code to point to? Preprint?

Typographic issues

1. Line 120-121. "approximate and typically too low" is awkward

2. Line 152. “treated” maybe “examined?”. Seems like the wrong word.
3. Line 241. Should be “indicate that, pooled”
4. Line 276. “opposed” seems like the wrong word.
5. Line 299-301. This sentence reads awkwardly.
6. Line 305. “exemplary” I think you mean “example”. worth rephrasing.
7. Line 308. “resulted in considerably too low” is awkward.
8. Line 355. “increase in parallel the later” is awkward.
