# Supplementary material for: Collaborative nowcasting of COVID-19 hospitalization incidences in Germany
Source: PLoS Comput Biol. 2023 Aug 11;19(8):e1011394. doi: 10.1371/journal.pcbi.1011394 (PMC10446237; doi:10.1371/journal.pcbi.1011394)
Supplement: Attachment — Submitted filename: Response.pdf [file pcbi.1011394.s003.pdf]

PCOMPBIOL-D-23-00600: Point-by-point response to the referees' comments

Daniel Wolffram et al.

18th July 2023

We would like to thank the review team for the constructive reports and helpful comments on our paper. In what follows we respond to the different points raised by the reviewers. The page references in the referees' statements refer to the original version. We also provide a version of the revised paper with track changes.

Response to Referee 1

The paper presents a comprehensive evaluation of real-time nowcasting methodologies as applied to COVID-19 hospitalisation incidence in Germany. The measurement of performance utilised appropriate standard scoring rules, with remarks made on the implications of these results on both public health communication and the statistical design of nowcasting models. In particular, the discussion of what model design decisions likely contributed to under- or over-performance is likely to be valuable for guiding future research. The paper is strengthened through its use of a preregistered study design and reproducible methodology. I note a few suggestions that could be made to improve the paper.

***1.1**: Addition of the word COVID-19 to the abstract would make the transition from general discussion of nowcasting to the paper's specific context clearer.*
Reply: Thank you for pointing this out. We now explicitly added COVID-19 to the abstract.

> *In this paper, we present a preregistered real-time assessment of eight nowcasting approaches, applied by independent research teams to German 7-day hospitalization incidences during the COVID-19 pandemic.*

1.2: *Figures 4 and 5 do not have years present on the x-axis or mentioned in the figure caption. The interpretation of this figure (especially regarding the emergence of Omicron) would be clearer with an explicit year present*
Reply: Thank you, we now added years to the x-axis. Note that the figures in question are now Figures 5 and 6.

1.3: *The effect of day of week on reporting delays is presented (Figure 2), with some models within the ensemble including this as a covariate (Table 1), however, there is little discussion of the impact of this effect on nowcasting performance. Given the large number of modelling problems where the inclusion of a day of week effect must be considered, reflection on this could be interesting.*
Reply: We agree that this is an interesting issue. We added a remark and Supplementary Figures A8–A10 to qualitatively illustrate the impact of weekday effects.

> *Additionally, stratified results by day of the week are shown in Fig A8 in S1 Appendix. For* `KIT` *and* `SZ`*, which did not account for weekday effects, we indeed observe performance differences for different weekdays. Fig A9 in S1 Appendix further shows nowcasts by* `KIT` *as issued on different weekdays, with a tendency for underprediction on Mondays and many cases of overprediction on Fridays or Saturdays. As seen in Fig A10 in S1 Appendix,* `ILM` *on the other hand did not exhibit any such differences by weekday.*

Response to Referee 2

This paper examines the results of a collaborative, preregistered, nowcasting experiment on German hospitalizations during a 6 month period of the COVID-19 pandemic. The analysis is thorough and carefully described, and the results are of general interest to the community.

2.1: *In section 2.1, the description of the target and reference date is rather complicated. I suggest adding a figure describing how to understand what this target is and how it relates to some of the issues discussed later in the manuscript.*
Reply: We have added Figure 2 to illustrate the nowcasting target as well as the *reference date* more clearly.

2.2: *Section 2.4. On the Ensembling, what happens if the resulting aggregated quantiles cross? Perhaps this never happened given the wide set of intervals, but it's worth thinking about, and mentioning as a potential issue. (Also, potentially for the individual models if they aren't derived from distributional forecasts). I believe one can show that sorting the quantiles is guaranteed to improve WIS, e.g if a forecast was (2, 1, 5, 7, 9, 15, 20) for the quantiles on line 150, simply sorting (1, 2, 5, 7, 9, 15, 20) is better.*
Reply: Thank you for raising this very good point. We were aware of this potential issue but mistakenly assumed our submission checks for the online repository would catch this. We added a remark on this issue.

We note that when using these aggregation approaches, quantile crossing can occur, meaning that the reported quantiles may not consistently increase with their nominal level [35]. To address this, one straightforward approach is to sort the quantiles in ascending order, which can improve the overall performance and coherence of the ensemble nowcasts [36]. Regrettably, this consideration was overlooked in our real-time ensemble, leading to some instances of quantile crossing. These were primarily caused by missing entries for a specific quantile level in one of the member models, as mentioned in Table A4 in S1 Appendix. As this occurred only in a small fraction of instances, we consider the impact on overall results negligible.

2.3: *In addition to the Relative Arithmetic Mean WIS described in section 2.5, I'd suggest reporting the Relative Geometric Mean WIS. There are a few reasons for this suggestion. (1) It's equivalent to the statistic in the paper, but using mean(log(WIS)) rather than WIS which is generally right skewed. (2) It has the simultaneous interpretation as the geometric Mean of WIS(m) / WIS(b), so that it can be seen as normalizing the error for a particular nowcast by degree-of-difficulty as given by the baseline. This latter interpretation also allows missing forecasts to be dropped without arbitrarily helping/hurting the overall score. (Now on looking at the Supplement, this is just a transformation of the pairwise comparison in C.1, right?)*
Reply: Thank you for this suggestion. Please note that there are some recent results that show that log(WIS) / log(CRPS) is not a proper scoring rule (see preprint by Bosse et al, *Transformation of forecasts for evaluating predictive performance in an epidemiological context*, `https://doi.org/10.1101/2023.01.23.23284722`, Section 2.4) for reference. The exact behavior of log(CRPS) does not seem to have been studied theoretically; however, the simulation example in Supplementary Figure SI.1 of the linked manuscript seems to imply that it favors overly sharp forecasts. We therefore refrained from using this measure.

2.4: *Section 3.3. It might be worth creating a figure to examine Relative WIS over time, rather than only over the whole period. It could go in the Supplement.*
Reply: Thanks for this suggestion. We provide a figure with Relative WIS over time in Supplementary Figure A11.

2.5: *Still section 3.3 (lines 254 and 262). It would be helpful here to have a sentence in each case that explains (or at least conjectures) why KIT has these deficiencies. In the second case, it seems plausibly due to the Max Delay (examined elsewhere), but it would be nice to at least point to that possibility here.*
Reply: In Section 3.3 we refrained from conjectures in order to keep the description and interpretation of results separate. In the following Section 3.4 we discuss some potential explanations and we have added a remark about the KIT model.

As can be seen from the diamond symbols in Fig 7 and in more detail from Fig A4 in S1 Appendix, the `KIT` *model did not issue particularly accurate*

point predictions (predictive medians). A likely reason is the lack of weekday effects, see Figs A8 and A9 in S1 Appendix. Its lower WIS values were primarily a result of better uncertainty quantification.

2.6: *The finding described on 292–93 strikes me as important. Maybe worth highlighting elsewhere as well? Seems like an important lesson for improving nowcasting models.*
Reply: We added a remark to the discussion in Section 4 to highlight this finding.

The exception was the `ILM` model which also stood out in terms of score-based performance for the national level and across age groups, and to a lesser degree the `KIT` model. These two models incorporated uncertainty quantification using past real-time nowcast errors which proved advantageous when compared to the standard model-based uncertainty intervals.

2.7: *Section 3.7 I don't quite understand the "rolling" approach as described. It would be good to do 1 or more of (a) give a concrete example, (b) explain mathematically, or (c) create a figure illustrating how to create this target.*
Reply: We agree that the different dates and targets are rather confusing. We added the respective formulas and Supplementary Figure A3 to explain this more clearly.

The original target allowed revisions until the final date of 8 August 2022, meaning that for different reference dates, adjustments could be made over time periods of different lengths (e.g., the hospitalization incidence for the first reference date in our study period could be completed over a longer time than that of the last reference date). Denoting the hospitalizations for reference date t reported with a delay of d days by $x_{t,d}$, the nowcasting target y_t can formally be written as

$$y_t = \sum_{i=0}^{6} \sum_{d=0}^{t_{\max}-(t-i)} x_{t-i,d} \quad \text{with } t_{\max} = \text{2022-08-08},$$

where the first sum represents a 7-day window ending in date t and the second sum accumulates the hospitalizations with reference date $t - i$ reported until $t_{\max}$.

An alternative to choosing one specific data version as the nowcast target is a "rolling" approach that considers delayed reports for each reference date t up to a specified maximum delay D. The nowcast target z_t then becomes

$$z_t = \sum_{i=0}^{6} \sum_{d=0}^{D} x_{t-i,d}.$$

In this case, the data for each reference date has the same amount of time to be revised. See Fig A3 in S1 Appendix for an illustration of these differently defined target time series.

2.8: *Given the success of* `ILM` *it would really help to have more detail about how it works. There's no reference or inclusion in Appendix D. So it reads as a blackbox that seems to do well. Is there at least a README or code to point to? Preprint?*
Reply: The code and a short explanation are now available in a GitHub repository and we referenced it in Section C of the Appendix.

Typographic issues

1. Line 120-121. "approximate and typically too low" is awkward
2. Line 152. "treated" maybe "examined?". Seems like the wrong word.
3. Line 241. Should be "indicate that, pooled"
4. Line 276. "opposed" seems like the wrong word.
5. Line 299-301. This sentence reads awkwardly.
6. Line 305. "exemplary" I think you mean "example". worth rephrasing.
7. Line 308. "resulted in considerably too low" is awkward.
8. Line 355. "increase in parallel the later" is awkward.
Reply: Thank you for pointing out these issues. We have addressed all of them.
